# Comparison of two accelerated 4D-flow sequences for aortic flow quantification

**DOI:** 10.1038/s41598-019-45196-x

**Published:** 2019-06-14

**Authors:** Sebastian Ebel, Josefin Dufke, Benjamin Köhler, Bernhard Preim, Susan Rosemeier, Bernd Jung, Ingo Dähnert, Philipp Lurz, Michael Borger, Matthias Grothoff, Matthias Gutberlet

**Affiliations:** 1 Department of Diagnostic and Interventional Radiology, University of Leipzig – Heart Centre, Leipzig, Germany; 2 Department of Simulation and Graphics, University of Magdeburg, Magdeburg, Germany; 3 Department of Diagnostic, Interventional and Paediatric Radiology, University of Bern, Bern, Switzerland; 4 Department of Paediatric Cardiology, University of Leipzig – Heart Centre, Leipzig, Germany; 5 Department of Cardiology, University of Leipzig – Heart Centre, Leipzig, Germany; 6 Department of Cardiac Surgery, University of Leipzig – Heart Centre, Leipzig, Germany

**Keywords:** Medical research, Cardiology

## Abstract

To compare two broadly used 4D-flow- with a 2D-flow-sequence in healthy volunteers, regarding absolute flow parameters, image quality (IQ), and eddy current correction (ECC). Forty volunteers (42 ± 11.8 years, 22 females) were examined with a 3T scanner. Thoracic aortic flow was assessed using a 3D-T2w-SPACE-STIR-sequence for morphology and two accelerated 4D-flow sequences for comparison, one with *k-t* undersampling and one with standard GRAPPA parallel-imaging. 2D-flow was used as reference standard. The custom-made software tool *Bloodline* enabled flow measurements for all analyses at the same location. Quantitative flow analyses were performed with and without ECC. One reader assessed pathline IQ (IQ-PATH) and occurrence of motion artefacts (IQ-ART) on a 3-point grading scale, the higher the better. *k-t* GRAPPA allowed a significant mean scan time reduction of 46% (17:56 ± 5:26 min vs. 10:40 ± 3:15 min) and provided significantly fewer motion artefacts than standard GRAPPA (IQ-ART 1.57 ± 0.55 vs. 0.84 ± 0.48; p < 0.001). Neither 4D-flow sequence significantly differed in flow volume nor peak velocity results with or without ECC. Nevertheless, the correlation between both 4D-flow sequences and 2D-flow was better with ECC; the *k-t* GRAPPA sequence performed best (R = 0.96 vs. 0.90). *k-t* GRAPPA 4D-flow was not inferior to a standard GRAPPA-sequence, showed fewer artefacts, comparable IQ and was almost two-fold faster.

## Introduction

Cardiovascular diseases (CVD) are the leading cause of death worldwide and a significant economic burden^1,2^. Therefore, precise evaluation of the cardiovascular system and the prediction of CVD are crucial. Two-dimensional (2D) phase contrast (PC) magnetic resonance imaging (MRI) (termed 2D-flow) enables non-invasive measurements and absolute quantification of flow, shunt volumes and flow velocities^3^.

Time-resolved, three-directional, three-dimensional (3D) PCMRI (termed 4D-flow) with cardiac and respiratory gating is a technique for measuring flow with full coverage of complete vascular systems, such as the great mediastinal vessels^4^. In addition to absolute quantification, 4D-flow gives new insights into physiologic and pathophysiologic flow patterns^5,6^.

4D-data acquisition still takes time. The most commonly used navigator-gated sequences require a rather long average acquisition time, which might be one reason, why 4D-flow is still not used as a standard method in clinical routines. In addition, long acquisition times may also cause more motion artefacts. Technical advances such as parallel imaging, advanced respiratory gating and strategies of undersampled acquisition allow reducing scan time while preserving image quality^7,8^.

The **G**ene**R**alized **A**utocalibrating **P**artially **P**arallel **A**cquisition (GRAPPA) is a parallel-imaging technique with linear interpolation of missing data in the *k-*space^9^.

More advanced parallel-imaging methods, including spatio-temporal undersampling with interpolation of missing data in the *k-t*-space, such as *k-t* GRAPPA, allow further acceleration of data acquisition^10–12^. Recently it has been shown that *k-t* GRAPPA accelerated 4D-flow sequences have the advantage of reduced scan time in the aorta and liver vasculature^13–15^. However, all these studies lack a comparison with 2D-flow as the gold standard in clinical MR protocols.

Furthermore, eddy currents can alter the characteristics of magnetic gradients, resulting in spatial and temporal phase offset that can compromise the accuracy of acquired flow data^16^, but it has not been assessed yet, if that error is different with various 4D-flow sequences.

Therefore, the purpose of this study was to compare two broadly used 4D-flow sequences with a 2D-flow sequence as the clinical gold standard. Furthermore, we wanted to prove the non-inferiority of a fast *k-t* GRAPPA accelerated 4D-flow sequence with a standard GRAPPA 4D-flow sequence. Additionally, we assessed background phase correction (eddy current correction - ECC) in all used sequences. A new, comprehensive, custom-made software tool was used for this evaluation, which allowed us to measure in the 4D-flow data sets always at the exact same level as our gold standard. In addition to the quantification of flow parameters, image quality, and susceptibility to artefacts were assessed.

## Material and Methods

### Study cohort

Datasets of 40 healthy volunteers with no history of CVD (22 females, mean age 41.8 ± 11.8 years) were included in the study. The local ethics board approved the study and written informed consent for use of the data was obtained from all participants. All experiments were performed in accordance with relevant guidelines and regulations^17,18^.

### MR image acquisition

All studies were performed using a 3 Tesla (T) scanner and a 16-channel anterior surface coil in combination with a 12-channel spine coil (Magnetom Verio Dot, Siemens Healthcare GmbH, Erlangen, Germany).

Before starting the MR-flow measurements, a high-resolution, T_2_-weighted single slab 3D TSE-Sequence (3D-T_2_-w-**SPACE**-STIR – **S**ampling **P**erfection with **A**pplication optimized **C**ontrasts using different flip angle **E**volution) with slab selective, variable excitation pulses and navigator respiratory control was acquired in coronal orientation covering the whole thorax for later standardized and uniform vessel segmentation^19^.

All flow measurements were performed with a constant encoded velocity (V_enc_) of 150 cm/s in all directions (4D) or in the direction perpendicular to the main flow vector (“through-plane”) in 2D-flow measurements.

The free-breathing 2D-flow acquisitions were performed first in a standardized manner in the mid-ascending aorta - by dividing the distance from the aortic valve to the brachiocephalic trunk - and in the descending aorta at the level of the left inferior pulmonary vein (LIPV) perpendicular to the centreline of the aorta. These data were used as the reference standard for the 4D-flow measurements.

The 4D-flow data were acquired in a sagittal oblique 3D volume covering the whole thoracic aorta. The first 4D-flow data were acquired using standard parallel imaging with undersampling along the phase encoding (k_y_) direction (GRAPPA) with an acceleration factor of 2, navigator gating, temporal resolution = 39.4 ms, spatial resolution = 2.5 × 2.5 × 2.5 mm^3^ ^9^. Variable imaging parameters such as the field of view (FOV) (300 mm^2^) and encoded phases (25) were kept constant in the *k-t* GRAPPA acquisition. Next, *k-t* accelerated 4D-flow data (undersampling along k_y_, k_z_ and t dimensions) with an acceleration factor 5, navigator gating, temporal resolution = 39.2 ms, spatial resolution = 2.5 × 2.5 × 2.5 mm^3^ were obtained as reported by Jung *et al*.^8,20^ (Table 1).

**Table 1:** Typical acquisition parameters of the different sequences in a healthy participant with a heart rate of 60 BPM.

	TE [ms]	TR [ms]	Voxel size [mm]	temporal resolution [ms]	bandwidth [Hz/Pixel]	FOV [mm²]	Flip Angle [°]	ECG-Gating	net acceleration factor
2D-flow	2.85	20.5	1.9 × 1.9	20.48	453		15	retrospective	—
4D-flow GRAPPA	2.48	38.56	2.5 × 2.5 × 2.5	39.4	453	300	15	retrospective	1.7
4D-flow *k-t* GRAPPA	2.3	37.6	2.5 × 2.5 × 2.5	39.2	450	300	15	retrospective	4.3
3D T2w-SPACE-STIR	99	2200	1.1 × 1.1 × 2	2200	651	300	150	none	—

Abbreviations: TE = echo time; TR = repetion time; ECG = electrocardiogram; 2D-/4D-flow = 2-/4-dimensional flow imaging.

### Cardiac magnetic resonance (CMR) data analysis

#### Vessel segmentation, blood flow visualisation and preprocessing

All processing and measurement steps were carried out using the custom-made software tool *Bloodline*^21,22^ (Department of Simulation and Graphics, University of Magdeburg, Germany). Anatomical 3D reconstruction of the aorta was derived from the 3D-T2w-SPACE-STIR sequence. *Bloodline* enables the import of pre-segmented data from different sequences. Therefore, the same segmented dataset could be used for both 4D-flow acquisitions and the reconstruction of the “simulated” 2D-flow measurements at the same level as the reference standard to ensure a standardized and uniform comparison. A centreline was semiautomatically drawn through the whole thoracic aorta beginning at the level of the aortic root. Intraaortic blood flow was visualized using time-resolved pathlines. We corrected for phase wraps, eddy currents and background noise as reported previously. Eddy current correction (ECC) was performed using a background subtraction technique^22,23^, in which an area of static tissue was semiautomatically defined, and the mean phase information of this area was subtracted from the ROI of the vessel.

### Measurements and flow quantifications

**Figure 1:**
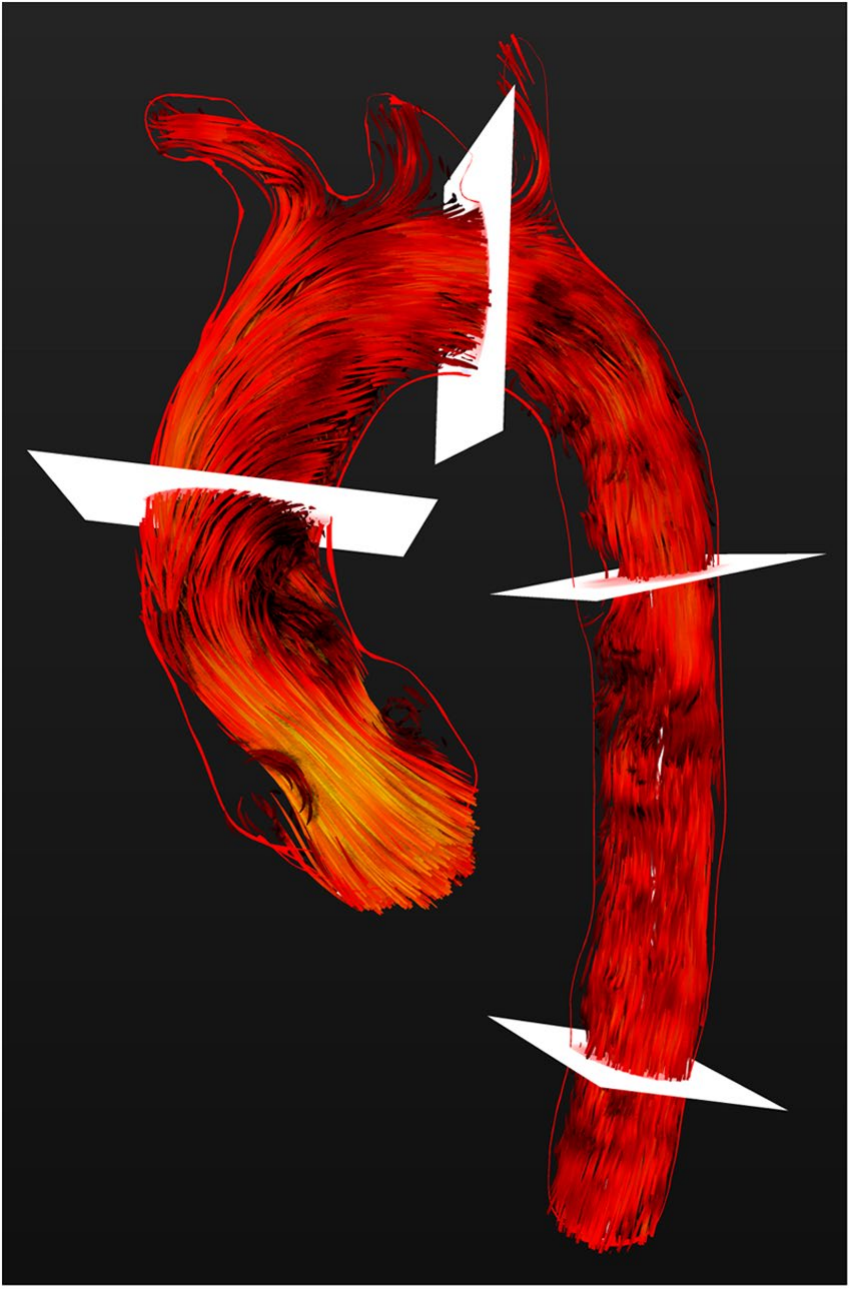
Visualization of systolic intraaortic blood flow with time-resolved 3D pathlines (red). The red rim represents the VRT of the thoracic aorta calculated from a 3D-T2w SPACE- STIR-sequence. Measuring planes in the ascending aorta, aortic arch, descending aorta and on the level of the diaphragm are depicted in white.

Measurements were carried out by one observer with more than 4 years of experience in cardiac MR (CMR) using *Bloodline*. 2D- and 4D-flow measurements were performed in a randomized order to guarantee a blinded reading. For 4D-flow quantification of the flow volume (ml/cycle) and peak velocity (m/sec), measuring planes were positioned at specific landmarks (Fig. 1) as follows:

**Plane 1**: Mid-ascending aorta (AAo) - same level as the 2D-flow [halved distance aortic valve - brachiocephalic trunk]

**Plane 2**: Aortic arch (AArch) right before the origin of the left subclavian artery

**Plane 3**: Descending aorta (DAo) at the same level as the 2D flow

**Plane 4**: Descending aorta at the level of the diaphragm (ADia).

All measurements were performed in segments without occurrence of “ghosting artefacts”. All measuring planes were oriented perpendicular to the centreline of the thoracic aorta (Fig. 1). All measurements were carried out twice, namely, with and without ECC.

#### Image quality

The image quality (IQ) regarding the reconstructed pathlines (IQ-PATH) of both 4D-flow datasets was assessed visually by cine pathline evaluation using a modified 3-point grading scale introduced by Schnell *et al*.^13^ as follows:

**0 – no filling**: no visible pathlines in the thoracic aorta

**1 – incomplete filling**: pathlines reached the aortic arch

**2 – complete filling**: pathlines in the descending aorta.

**Figure 2:**
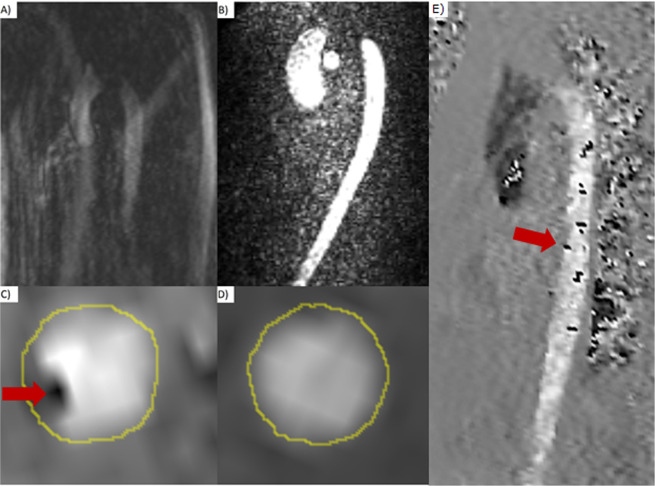
(**A**) Magnitude image of the 4D-flow sequence with GRAPPA showing breathing-related motion artefacts in the whole field of view. (**B**) Magnitude image of the 4D-flow sequence with *k-t* GRAPPA showing no artefacts. (**C**) Phase image of the 4D-flow sequence with GRAPPA showing a cross section through the descending aorta. In the left lower quadrant, one can see an artefact with an “aliasing-like” appearance (marked with red arrow), although the peak velocity never exceeded the maximum encoded velocity. (**D**) Phase image of the 4D-flow sequence with *k-t* GRAPPA showing a cross section through the descending aorta in the same volunteer at the exact same level shown in (**C**) with no artefact. (**E**) “In-plane” phase image of the 4D-flow sequence with GRAPPA of the same volunteer demonstrating several artefacts in the descending aorta with an “aliasing like” appearance. The red arrow indicates the level of the cross sectional phase image in (**C**).

Regarding motion, breathing (Fig. 2) or aliasing artefacts (IQ-ART), we analysed the reconstructed magnitude and phase images of both 4D-flow sequences using a 3-point grading scale as follows:

**0 –** artefacts within the aorta

**1 –** artefacts outside the aorta

**2 –** no visible artefacts.

### Statistical analysis

For volunteer characteristics, all results were given as their mean values and standard deviation. All analyses were performed using MedCalc Statistical Software V15.11.4 (MedCalc Software, Ostend, Belgium). To check whether the data were normally distributed, a Shapiro-Wilks test was performed. Once normality was proven, a paired t-test for net flow, peak velocity or image quality was performed. A p-value < 0.05 was considered statistically significant. Correlation analyses were performed with interclass-correlation, scatter analysis and Bland-Altman plots. Bland-Altman analysis provided the mean differences between measurements (bias), the standard deviation of the mean (SD) and the limits of agreement (LOA) used for the different approaches of flow analysis.

### Ethics approval and consent to participate

Local ethics board approved this study: Ethik-Kommission an der Medizinischen Fakultät der Universität Leipzig AZ 443/16-ek.

## Results

### Acquisition time

**Figure 3:**
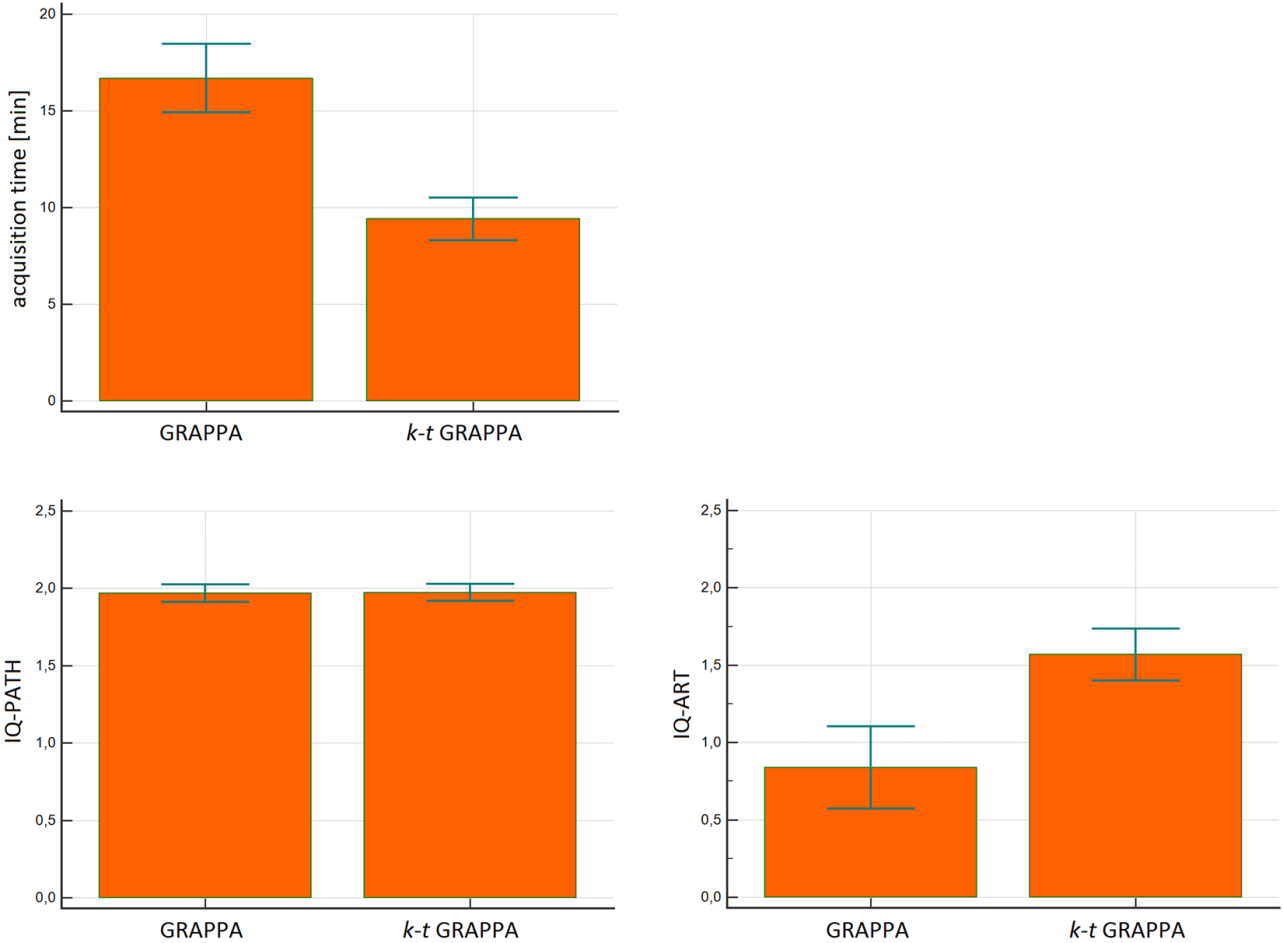
Box-plot comparison of (**A**) the mean acquisition time (min) of the GRAPPA accelerated versus the *k-t* GRAPPA accelerated 4D-flow sequence and the corresponding image quality (IQ) regarding (**B**) pathline filling (IQ-PATH) and (**C**) motion, breathing and aliasing artefacts (IQ-ART). Significant differences are marked by ***n.s = not significant.

Complete data sets were successfully acquired in all 40 participants. No adverse events occurred. Mean acquisition time was 17:56 min (±5:26 min) for GRAPPA and 9:40 min (±3:15 min) for *k-t* GRAPPA. This difference was statistically significant (*p* = 0.002) (Fig. 3). A mean scan time reduction by 46.12% was achieved for *k-t* GRAPPA compared to GRAPPA.

### Image quality and artefacts

IQ-PATH assessment demonstrated almost complete pathline filling in the descending aorta in all volunteers for both sequences. IQ-PATH of the 4D-flow GRAPPA and *k-t* GRAPPA sequence showed comparable results with an excellent mean IQ-score of 1.97 (±0.16) and 1.98 (±0.15), respectively with no significant differences between the measurements.

IQ-ART - regarding motion, breathing and aliasing artefacts - demonstrated significant differences (*p* < 0.001) with a mean value of 0.84 ± 0.48 for GRAPPA and higher IQ-ART values of 1.57 ± 0.55 for the *k-t* GRAPPA sequence.

Almost all artefacts could be characterized as ghosting artefacts by analysing the reconstructed magnitude images due to breathing-related motion of the thoracic wall as described previously^20^. In the reconstructed phase images, especially in the feet-head direction, the artefacts looked similar to aliasing (Fig. 2C,E), but according to the 2D-flow measurements in the same direction it could be verified, that the V_enc_ of 150 cm/s was never exceeded. In contrast to “real” aliasing artefacts, the ghosting artefacts appeared randomly scattered across the vessel and were not grouped. By looking at the magnitude images, ghosting artefacts could be distinguished from real aliasing. Furthermore, the inherent phase wrap correction algorithm of *Bloodline* was not triggered by these artefacts and therefore did not wrongly correct for these artefacts. Visually, these artefacts occurred more often in the descending aorta (Fig. 2E).

### Flow quantification

**Figure 4:**
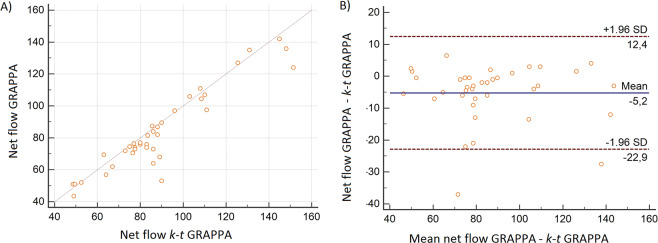
Comparison of 4D-flow measurements in the AAo: (**A**) Linear regression analysis of the net flow (ml/cycle) as measured with the GRAPPA- and *k-t* GRAPPA accelerated 4D-flow sequence with a good correlation and a correlation coefficient of r = 0.94; p < 0.0001 and (**B**) the corresponding Bland-Altman plot demonstrated good limits of agreement (LOA) with −22.9 to 12.4 of the net flow (ml/cycle).

The mean net flow in the AAo of the GRAPPA sequence was slightly, but not significantly (*p* = 0.782), lower compared to the *k-t* GRAPPA sequence (83.54 ml/cycle (±25.03) vs. 88.14 ml/cycle (±26.2), R = 0.94) (Fig. 4A). Bland-Altman analysis showed a mean difference between measurements of only 5.2 ml/cycle (Fig. 4B). The mean peak velocity showed a moderate correlation with no significant differences (p = 0.234) between both sequences (1.21 m/sec (±0.29) vs. 1.18 m/sec (±0.31), R = 0.6). Bland-Altman analysis showed a mean difference between measurements of 0.02 m/sec, and LOA was −0.61–0.64 m/sec. We found no significant differences between the two 4D measurements in the AArch, Dao or ADia regarding net flow and peak velocity (Table 2).

**Table 2:** Distribution of measurements of mean net flow and peak velocity in the different 4D flow sequences GRAPPA and *k-t* GRAPPA in different measuring planes with p-value, correlation coefficient and limits of agreement.

	Net flow [ml/cycle]				peak velocity [m/sec]			
	GRAPPA [ml/cycle]	*k-t* GRAPPA [ml/cycle]	Correlation coefficient R/p-value	Limits of agreement (LOA)	GRAPPA [ml/cycle]	*k-t* GRAPPA [ml/cycle]	Correlation coefficient R/p-value	Limits of agreement (LOA)
Ascending Aorta	83.54 (±25.03)	88.14 (±26.21)	0.94/<0.0001	−22.9–12.4	1.21 (±0.29)	1.18 (±0.31)	0.60/<0.0001	−0.61–0.64
Aortic Arch	67.34 (±20.75)	68.5 (±21.19)	0.92/<0.0001	−18.6–15.1	1.1 (±0.32)	1.01 (±0.29)	0.65/<0.0001	−0.42–0.64
Descending Aorta	50.26 (±17.12)	49.56 (±17.38)	0.87/<0.0001	−17.3–17.9	1.0 (±0.34)	0.93 (±0.44)	0.91/<0.0001	−0.31–0.44
Descending Aorta - level of diaphragm	42.37 (±18.76)	40.1 (±18.6)	0.83/<0.0001	−21.7–24.2	1.06 (±0.35)	0.92 (±0.34)	0.55/ <0.0001	−0.52–0.78

The given p-values refer to the significance of the correlations.

### Validation of 4D- against 2D-flow as the reference standard

No significant differences regarding the net flow or peak velocities occurred in the AAo and DAo when comparing both 4D-flow measurements with the reference standard 2D-flow at the exact same position (Detailed results in Table 3).

**Table 3:** Comparison of the mean net flow **[ml/cycle]** and peak velocity **[m/sec]** calculations to 2D-flow before and after performing eddy current correction (ECC) in GRAPPA- and *k-t* GRAPPA-accelerated 4D-flow measurements with p-values and correlation coefficients.

	Net flow [ml/cycle]						Peak velocity [m/sec]					
	Without ECC			With ECC			Without ECC			With ECC		
	4D-flo w GRAPPA	2D-flow	Correlation coefficient R/p-value	4D-flow GRAPPA	2D-flow	Correlation coefficient R/p-value	4D-flow GRAPPA	2D flow	Correlation coefficient R/p-value	4D-flow GRAPPA	2D flow	Correlation coefficient R/p-value
Ascending Aorta	80.36 (±26.25)	87.86 (±24.26)	0.84/<0.0001	83.54 (±25.03)	87.86 (±24.26)	0.90/<0.0001	1.25 (±0.34)	1.21 (±0.28)	0.46/<0.0001	1.21 (±0.29)	1.21 (±0.28)	0.87/<0.0001
Descending Aorta	50.01 (±20.81)	49.58 (±18.13)	0.77/<0.0001	50.26 (±17.12)	49.58 (±18.13)	0.85/<0.0001	1.15 (±0.42)	0.95 (±048)	0.53/<0.0001	1.0 (±0.34)	0.95 (±048)	0.89/<0.0001
	**4D-flow** ***k-t*** **GRAPPA**	**2D flow**	**Correlation coefficient R**/**p-value**	**4D-flow** ***k-t*** **GRAPPA**	**2D flow**	**Correlation coefficient R**/**p-value**	**4D-flow k** ***-t*** **GRAPPA**	**2D flow**	**Correlation coefficient R**/**p-value**	**4D-flow** ***k-t*** **GRAPPA**	**2D flow**	**Correlation coefficient R/p-value**
Ascending Aorta	87.41 (±26.01)	87.86 (±24.26)	0.94/<0.0001	88.14 (±26.21)	87.86 (±24.26)	0.98/ <0.0001	1.15 (±0.32)	1.21 (±0.28)	0.80/<0.0001	1.18 (±0.31)	1.21 (±0.28)	0.93/<0.0001
Descending Aorta	48.02 (±18.13)	49.58 (±18.13)	0.98/<0.0001	49.56 (±17.38)	49.58 (±18.13)	0.98/<0.0001	0.94 (±0.48)	0.95 (±048)	0.99/<0.0001	0.93 (±0.44)	0.95 (±048)	0.99/<0.0001

**Figure 5:**
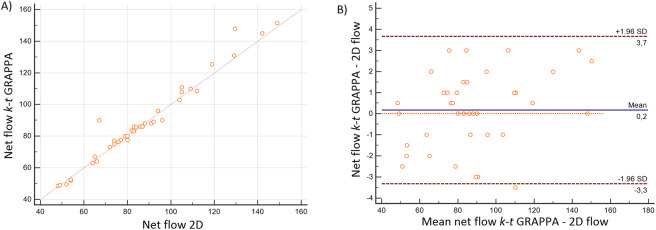
Comparison of measurements in 4D flow k-t GRAPPA and 2D flow in the AAo: (**A**) Linear regression analysis of the net flow (ml/cycle) as measured with the *k-t* GRAPPA- and 2D flow sequence with a correlation coefficient of r = 0.98; p < 0.0001 and (**B**) the corresponding Bland-Altman plot demonstrated good limits of agreement (LOA) with −3.3 to 3.7 of the net flow (ml/cycle).

However, the level of agreement (LOA) was substantially different between both 4D-flow sequences, from good correlations with r = 0.85–0.90 for the GRAPPA accelerated to very good correlations with R = 0.93–0.99 for the *k-t* GRAPPA sequence. Additionally, all correlations were statistically significant (p < 0.0001) (Fig. 5). Furthermore, good LOA could be demonstrated with both 4D-flow sequences, but the smallest LOA was with the *k-t* GRAPPA accelerated sequence (Table 3).

### Impact of eddy current correction

Despite no overall significant differences between the 4D-flow analysis with and without ECC, we found a general tendency to lower mean flow volumes and higher mean peak velocities without ECC.

The mean net flow in the AAo using the GRAPPA accelerated sequence was 80.36 ml/cycle (±26.25) without ECC and 83.54 ml/cycle (±25.03) with ECC. The mean difference between measurements was −3.09 ml/cycle (±6.49), which was not statistically significant (p = 0.77) (Table 4).

**Table 4:** Results of comparison of measurements of mean net flow and peak velocity before and after performing eddy current correction in 4D flow GRAPPA 2 and *k-t* GRAPPA 5 with p-value and correlation coefficient.

4D-flow *k-t* GRAPPA	Net flow			Peak velocity		
	Without ECC	With ECC	Mean difference without - with ECC (SD)	Without ECC	With ECC	Mean difference without - with ECC (SD)
Ascending Aorta	87.41 (±26.01)	88.14 (±26.21)	−0.71 (±3.45)	1.27 (±0.32)	1.18 (±0.31)	0.02 (±0.17)
Aortic Arch	64.85 (±21.09)	68.5 (±21.19)	−0.67 (±2.97)	1.02 (±0.34)	1.01 (±0.29)	0.04 (±0.10)
Descending Aorta	48.02 (±18.13)	49.56 (±17.38)	−0.65 (±2.33)	0.94(±0.48)	0.93 (±0.44)	0.01 (±0.09)
Descending Aorta - level of diaphragm	41.01 (±19.17)	40.1 (±18.6)	−0.78 (±3.01)	0.93 (±0.36)	0.92 (±0.34)	0.02 (±0.03)
**4D-flow GRAPPA**	**Without ECC**	**With ECC**	**Mean difference without - with ECC (SD)**	**Without ECC**	**With ECC**	**Mean difference without - with ECC (SD)**
Ascending Aorta	80.36 (±26.25)	83.54 (±25.03)	−3.09 (±6.49)	1.25 (±0.34)	1.21 (±0.29)	0.07 (±0.22)
Aortic Arch	67.2 (±21.65)	67.34 (±20.75)	−3.14 (±5.11)	1.1 (±0.32)	1.1 (±0.32)	0.14 (±0.11)
Descending Aorta	50.01 (±20.81)	50.26 (±17.12)	−2.16 (±9.05)	1.15 (±0.42)	1.0 (±0.34)	0.14 (±0.41)
Descending Aorta - level of diaphragm	43.41 (±19.22)	42.37 (±18.76)	−2.92 (±4.33)	1.08 (±0.32)	1.06 (±0.35)	0.2 (±0.33)

The mean peak velocity was 1.25 m/sec (±0.34) without ECC and 1.21 m/sec (±0.29) with ECC, with no significant differences (p = 0.45). The mean difference between measurements was −0.07 m/sec (±0.22).

The mean net flow in the AAo using the *k-t* GRAPPA accelerated sequence was 87.41 ml/cycle (±26.01) without ECC and 88.14 ml/cycle (±26.21) with ECC. The mean difference between measurements was −0.71 ml/cycle (±3.45), which was not statistically significant (p = 0.900) (Table 4).

The mean peak velocity was 1.27 m/sec (±0.32) without and 1.18 m/sec (±0.31) with ECC, with no significant differences (p = 0.624). The mean difference between measurements was −0.02 m/sec (±0.17).

We found no significant differences between measurements with and without ECC in all other measured areas (Table 4). However, when comparing 4D-flow measurements with the reference standard 2D-flow, we generally found slightly higher correlations between measurements with ECC (AAo GRAPPA: r = 0.90; *k-t* GRAPPA: 0.96) than without ECC (AAo: GRAPPA: r = 0.84; *k-t* GRAPPA: r = 0.94) regarding net flow, and even more substantial differences regarding peak velocities with GRAPPA (with ECC: r = 0.87 and without ECC: 0.46) and also k-t GRAPPA (0.93 vs. 0.80) (Table 3).

In summary, results of ECC did not differ significantly in the two used different acceleration techniques.

## Discussion

In this study, we demonstrated a significant scan time reduction for the *k-t* GRAPPA compared to the GRAPPA sequence, without loss in image quality. This finding is in line with previous studies that demonstrated the value of *k-t* acceleration in the assessment of the thoracic aorta and the liver vasculature and with the SCMR 4D-flow consensus statement^13,14,18^. Nevertheless, considering the net acceleration factors of the two 4D-flow sequences, there should be a time reduction of a factor of 2.5. One reason for a smaller time reduction of *only* 46% in our study could be a lower navigator efficacy due to unsteady breathing patterns of the volunteers during the acquisition of the *k-t* sequence (not depicted), which was always obtained at the very end of the examination.

The mean acquisition time for the whole thoracic aortic flow by *k-t* GRAPPA acceleration was 9:40 min. This might be already a reasonable acquisition time in a study setting scanning healthy volunteers, but not in a clinical setting with real patients who undergo cardiac MRI examinations, which usually already take 40–50 min. without 4D-flow acquisitions. Therefore, - so far only in a limited number of patients - further 4D-flow imaging strategies for acquisition time reduction have been described. Liu *et al*. showed in 3 healthy volunteers that an acceleration strategy based on time-resolved, variable-density random sampling allows scanning the thoracic aorta in under 5 minutes^24^. Other studies showed that it is possible to acquire 4D data of the aorta in less than two minutes^25,26^. In these early first studies, only small cohorts were included, therefore, further evaluations of these techniques and strategies for shortening 4D-flow scan time are needed. Other sources like Cheng *et al*. or Christodoulou *et al*. introduced different advanced acceleration techniques like *XD flow* and *MR multitasking* to shorten CMR acquisition time^27,28^. However, both proposed techniques need the administration of intravenous contrast agents. In the here presented study we used sequences with sufficient SNR even without the use of additional contrast agents. Despite the fact, that the application of MR contrast agents is generally safe, a minor risk of allergic reactions, extravasation or the so far not completely understood gadolinium-accumulation in the brain exists. Therefore, the authors state that imaging techniques without the need for contrast agents should be preferred^29^.

More importantly, we could demonstrate that *k-t* undersampling leads to a greater resistance to artefacts and therefore improves diagnostic accuracy and better correlates with the current standard of care – 2D-flow measurements. In 2007, Markl *et al*. showed that navigator-gating can improve diagnostic accuracy and reduces blurring and ghosting artefacts^20^. This finding is in line with the consensus statement from 2015 recommending the use of respiratory motion compensation with a navigator on the liver/diaphragm interface^16^. Although both sequences we used in our study were navigator-gated, GRAPPA showed a significant higher susceptibility to breathing-related artefacts compared to *k-t* GRAPPA.

Therefore, we conclude that longer acquisition times with more respiratory cycles increase the burden of breathing-related artefacts. In other words, shorter acquisition times cause higher resistance to motion artefacts. We showed that these artefacts have the same appearance as aliasing in phase contrast images, although the maximum encoded velocity was never exceeded, and phase wrap correction had no impact on these artefacts. These artefacts occurred more frequently in the descending aorta. This – in line with other sources – highlights the importance of careful quality control of 4D-flow data sets for every clinical and research study before performing any measurements^18,30–32^.

In this study, we used the software *Bloodline* for processing and analysing the 4D-flow data, which enables measurements in different 4D-flow sequences from the same participant at the exact same spot, so we could guarantee that the comparison between both 4D sequences and 2D-flow was performed under the same equal conditions. Both 4D-flow sequences show good agreement regarding net flow and peak velocity in all investigated measuring planes. This finding is in line with previous studies^13^; here, the authors found a strong agreement between a standard GRAPPA accelerated 4D-flow sequence and a 4D-flow sequence with *k-t* undersampling, with a mean net flow of 88.3 ml/cycle in both sequences.

Compared to previous studies we added a validation against a 2D-flow sequence as the clinical gold standard, and included a larger cohort of healthy volunteers.

In 2014, Giese *et al*. showed a good correlation (up to R = 0.93) between a *k-t* accelerated 4D-flow sequence and 2D-flow (regarding flow volumes and velocities in the ascending aorta) in 10 healthy volunteers and in patients with congenital heart disease using a 1.5 T scanner^33^. They found a slight underestimation of peak velocities in the 4D-flow sequence used compared to 2D-flow. In our study, we found no such differences. Hanneman *et al*.^34^ compared pulmonary to systemic flow in 4D- and 2D-flow and found a mediocre correlation (R = 0.67) regarding net flow in the ascending aorta; by comparison, we found R = 0.9 (GRAPPA) and R = 0.96 (*k-t* GRAPPA). Possible reasons for these differences could be that they used a 1.5 T scanner and a 4D-flow sequence without parallel imaging, and we used 3 T scanner and 4D-flow sequences with GRAPPA and *k-t* GRAPPA.

Imaging at higher field strengths improves image quality by a higher SNR, allowing the use of faster imaging, which additionally reduces the amount of motion artefacts.

In a final step, we elucidated the impact of eddy current correction on the measurements of net flow and peak velocity, and we found no significant differences between measurements and no differences in comparison to 2D-flow, so that we conclude that ECC does not depend on the used acceleration technique. However, with ECC, we found a substantially better correlation between both 4D-flow sequences with 2D-flow than without ECC, although there were no significant differences.

This fits with findings of Ballache *et al*.^35^. They also found no significant differences regarding flow measurements in the aorta with and without ECC, although they found a high sensitivity to eddy currents in measurements in the ascending aorta; therefore, the authors highlighted the importance of ECC. In the literature, there is consent that in 4D-flow measurements an ECC is necessary. However, there are currently many different techniques of ECC. In our work, we used a technique with background subtraction introduced by Bock *et al*.^23^. Other groups used similar but not identical strategies of ECC^36^; in this study, a phantom-based ECC technique was performed, and the group showed that the severity of errors caused by eddy currents depends on the position of the measured vessel relative to the magnetic iso-centre. Additionally, the severity of eddy currents depends on many different parameters, including scanner type and Venc and even temperature^37^. The diversity of results in the literature highlights the need for a study that systematically evaluates the advantages and disadvantages of different ECC strategies. To the best of our knowledge, there is no such study yet. In conclusion, we agree with Lotz *et al*. that ECC is necessary but should be used with great caution, since it is not clear which technique provides accurate data and which technique introduces more errors than it compensates for^38^.

The limitations of this study are that we only compared a 4D-flow sequence with *k-t* undersampling with one sequence without *k-t* undersampling, but we did not perform a systematic evaluation of the impact of different *k-t* acceleration factors. However, the aim of this study was to evaluate which 4D-flow sequence is more suitable for use in further studies, and not to compare different *k-t* undersampling strategies as was done previously^8,13,14^. Furthermore, we only used one approach for ECC and did not perform a systematic analysis of different correction methods; this has to be addressed in further studies.

In conclusion, we demonstrated in a large cohort of 40 volunteers that 4D-flow with *k-t* undersampling provides reliable and accurate flow data that are as good as 4D-flow without *k-t* acceleration. Both sequences agreed strongly with the current standard of care 2D-flow. *k-t* GRAPPA outperformed GRAPPA in terms of acquisition time and resistance to artefacts. Although we found no significant differences between measurements of net flow and peak velocity with and without ECC, there is a better correlation with 2D-flow after correction for eddy currents. The authors conclude that it is justifiable to use 4D-flow sequences with *k-t* undersampling for future research projects.

## Data Availability

Please contact author for data requests.
